# A Fast Verified Liveness Analysis in SSA Form

**DOI:** 10.1007/978-3-030-51054-1_19

**Published:** 2020-06-06

**Authors:** Jean-Christophe Léchenet, Sandrine Blazy, David Pichardie

**Affiliations:** 8 CNRS, LIG, Université Grenoble Alpes, Saint Martin d’Hères, France; 9 University Koblenz-Landau, Koblenz, Germany; Univ Rennes, Inria, CNRS, IRISA, Rennes, France

**Keywords:** Liveness analysis, SSA form, Dominance, Verified compilation

## Abstract

Liveness analysis is a standard compiler analysis, enabling several optimizations such as deadcode elimination. The SSA form is a popular compiler intermediate language allowing for simple and fast optimizations. Boissinot et al. [7] designed a fast liveness analysis by combining the specific properties of SSA with graph-theoretic ideas such as depth-first search and dominance. We formalize their approach in the Coq proof assistant, inside the CompCertSSA verified C compiler. We also compare experimentally this approach on CompCert’s benchmarks with respect to the classic data-flow-based liveness analysis, and observe performance gains.

## Introduction

In order to be precise, several important compiler analyses need to know the lifetime of variables. This is of course the case with deadcode elimination and register allocation, but also for instance with software pipelining and trace scheduling. Computing this information efficiently is thus of utmost importance. This is the purpose of *liveness analysis*.

Given a program and a variable, liveness analysis consists in determining the points of the program where this variable is needed, i.e. the points from which an execution can reach an instruction where this variable is used. At such points, this variable is said to be *live*. Like many other semantic properties, this property is undecidable and is classically over-approximated by its syntactic counterpart which considers, instead of real executions, paths in the control flow graph (CFG) of the program.

Traditionally, liveness information is computed by a backward data-flow analysis that computes monolithically the liveness status of all program variables at all program points [2]. In 2008, Boissinot et al. [7] described another method to compute this information, with two particularities. Firstly, their technique is applicable only to programs in SSA form, an intermediate language adopted by most of the modern compilers, e.g. LLVM [11]. Indeed, their approach relies on one of the key properties of SSA, that they combine with graph-theoretic notions. Secondly, it is not designed to compute the whole liveness information of the program, but instead to answer so-called liveness queries, of the form “is variable a live at point *q*?”. They call this approach, considering only one variable and one program point at a time, “liveness checking”. Since this approach computes only limited information compared to the data-flow based one, they claim that it outperforms it as long as the number of asked queries is low, which their experiments confirm.

In this paper, we focus on liveness checking, as presented in [7], from the point of view of formally verified compilation. In this context, an implementation of liveness checking should not only be efficient, as usual in compilation, but also needs to be formally proved correct.

We tackle this problem in the context of CompCert [12, 13], a verified C compiler written in the Coq proof assistant, and its fork with an SSA middle-end, CompCertSSA [3]. CompCert and CompCertSSA already contain several liveness analyses (e.g. in module  ), but all of them, like in the other verified compilers (e.g. CakeML [15]), are data-flow based. Our goal is to implement liveness checking on the SSA form of CompCertSSA, taking into account the particularities of Coq and CompCertSSA, and carefully enough so that we observe the expected performance improvement w.r.t. the data-flow based approach.

After describing liveness checking, as well as the required background, in detail in Sect. 2, we present the following contributions:

- an implementation of liveness checking in CompCertSSA (Sect. 3) adapting the ideas of [7] to CompCertSSA, including some advanced optimizations;
- a proof of correctness of this algorithm (Sect. 4) showing the validity of Boissinot et al.’s subtle graph-theoretic arguments;
- experiments on CompCert’s benchmarks (Sect. 5) showing that two variants of the liveness checking algorithm compare favorably w.r.t. the data-flow based approach.

The formalization and the experiments are available online [1].

## Background

We first recall some notions from graph theory and compilers in Sect. 2.1, then we give the idea of liveness checking in Sect. 2.2, before describing it in detail in Sects. 2.3 and 2.4.

### Basic Concepts in Graphs and Compilers

**Fig. 1.**
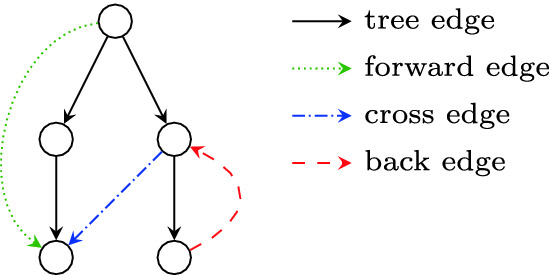
Example of edge classification

**Depth-First Search.** DFS classifies the edges of a graph into four categories (cf. Fig. 1): the *tree edges* that form a spanning tree, the *forward edges* connecting a node to one of its descendants in the spanning tree, the *cross edges* connecting a node to an unrelated node in the spanning tree, and the *back edges* connecting a node to one of its ancestors in the spanning tree.

**Fig. 2.**
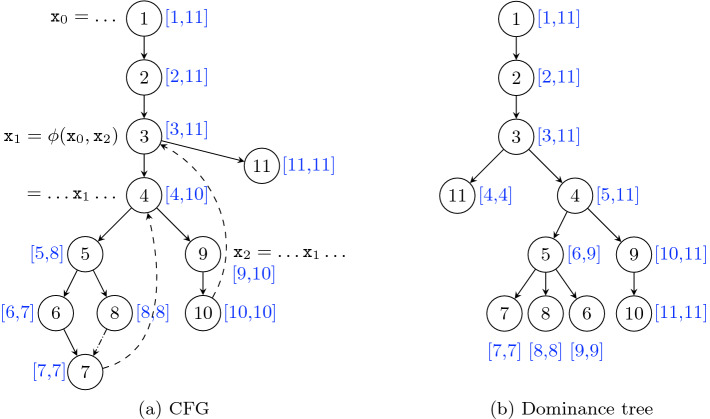
The CFG and dominance tree of an SSA program, both labeled with reachability intervals based on preorder numberings

**Encoding Reachability in a Tree.** It is possible to label each node of a tree with a pair of integers, allowing to determine whether a node is an ancestor of another node just by comparing their labels. One possible labeling is based on a DFS preorder numbering, the first integer of a node being its preorder number and the second one being the maximum preorder number in the subtree rooted at that node. An example of such a labeling is provided in Fig. 2b.

**Dominance.** The dominance relation is traditionally defined on a flow graph, i.e. a graph with a distinguished node *entry* such that every vertex is reachable from that node. We say that a node *u* dominates a node *v* if every path from *entry* to *v* goes through *u*; *u* strictly dominates *v* if *u* dominates *v* and *u* and *v* are distinct. Dominance is an order relation, i.e. it is reflexive, transitive and antisymmetric. Moreover, each node *u* distinct from *entry* has a unique strict dominator dominated by all the strict dominators of *u*, showing that dominance can be encoded as a tree, called the *dominance tree*.

**SSA Form.** The SSA form, standing for *Static Single Assignment*, is a program representation where each variable is textually defined at most once. To turn a non-SSA representation into SSA, variables that are assigned to multiple times are renamed so that each renamed version is associated to one definition point only. When two flows of the program, carrying two different versions of the same initial variable, merge at a so-called *join point*, we need a way to express which version is selected. SSA introduces special nodes for this, called $\phi$-nodes. The $\phi$-function inside the $\phi$-node takes as many arguments as the number of predecessors of the node. When the flow comes from the $i^{th}$ predecessor, the $\phi$-function returns the $i^{th}$ argument, thus selecting the version of the variable corresponding to that predecessor. $\phi$-nodes must be handled with care in terms of where they use and define variables. In this paper, each argument of a $\phi$-function is considered used at the corresponding predecessor of the $\phi$-node. The variables defined by the $\phi$-node are treated normally. An example SSA program is shown in Fig. 2, along with its dominance tree.

A program in *strict* SSA form is a program where each use of a variable is preceded by its definition (unique per definition of SSA). A program in strict SSA form obeys the *dominance property* [7], stating that each use of a variable is dominated by its definition.

**Liveness.** In this paper, by “live”, we mean “live-in”, which in the context of a program in strict SSA form can be defined as follows. A variable a is live-in at point *q* if there exists a path in the CFG from *q* to a use of a that does not go through the definition of a.

### Liveness Checking

Boissinot et al.’s algorithm answers liveness queries efficiently based on some precomputed information. The algorithm is thus composed of two parts: a precomputation part that captures information about the CFG structure and an online part that answers the liveness queries based on this information.

This architecture has two main advantages compared to the classic one. Firstly, the precomputation step is faster than the full liveness analysis. Thus, if the number of queries is rather small, this algorithm is faster than the classic one. Secondly, since the precomputation step depends on the CFG structure and not on liveness information, its result remains correct if the program is modified by some transformations that preserve its structure. In this sense, precomputed information is more robust than the liveness one.

Actually, the classic liveness analysis approach can also be seen as being made of a precomputation part (the analysis), followed by an online part (reading in the liveness table). From this point of view, Boissinot et al.’s algorithm just chooses a different trade-off than the classic approach: a faster precomputation at the cost of slower queries. As mentioned above, this compromise is interesting if the number of queries is low.

### Precomputation

Let us consider the following liveness query: “is variable a live at point *q*?”. This query amounts to checking whether a path exists between *q* and a use of a that does not go through the definition *d* of a. Note that, by the dominance property, we know that all uses of a are dominated by *d*. It is possible that a is used at *d*, but since in this paper by “live” we mean “live-in”, such a case has no impact on the answer to the query. We can thus restrict ourselves to the uses of a that are strictly *d*-dominated.

Let $\pi$ be a path from *q* to a use *u* of a that does not go through *d*. If there is a node *x* on $\pi$ that is not strictly *d*-dominated, we can show that *u* is not dominated by *d*, contradicting the dominance property. Reciprocally, a strictly *d*-dominated path from *q* to *u* does not go through *d*. This shows that a is live at *q* if and only if there exists a strictly *d*-dominated path from *q* to a use of a.

**Fig. 3.**
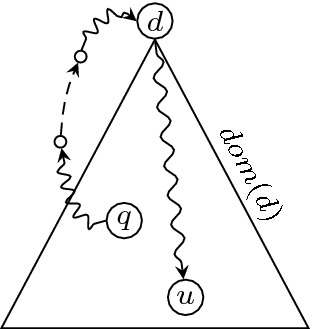
Leaving and reentering the set of strictly dominated nodes requires a back edge.

Boissinot et al. show more. If *q* is strictly *d*-dominated, any non-strictly *d*-dominated path from *q* to a use *u* of a goes through *d*, since it reenters the set of strictly dominated nodes, and the part of the path from *q* to *d* contains a back edge (intuitively, we need to go back up from *q* to *d*), represented as a dashed arrow in Fig. 3. Stated in the opposite way, if there exists a path from *q* to *u* that does not contain a back edge, then the path is strictly *d*-dominated, which shows that a is live at *q*.

Based on this observation, Boissinot et al.’s main idea is that back edges must be dealt with separately from the other edges. They suggest to decompose the reachability in the original graph into two relations, called *R* and *T*. Relation *R* captures the reachability in the reduced graph $\widetilde{G}$, the acyclic graph obtained by removing the back edges from the original graph. Relation *T* associates to each program point both itself and a set of interesting back edge targets.

Formally, *T* is the reflexive and transitive closure of $T^\uparrow$, where $T^\uparrow _t$ (cf. Definition 1)^1^ is the set of back edge targets not reduced reachable (i.e. reachable in the reduced graph) from node *t* but whose source is reduced reachable from *t*. For instance, in Fig. 2a, $T^\uparrow _5 = \{ 4 \}$, $T^\uparrow _4 = \{ 3 \}$, and thus $T_5 = \{ 3, 4, 5 \}$.

#### Definition 1

**(**$$T^\uparrow $$ **and** *T***).**

$$ T^\uparrow _t = \{ t' \in V \setminus R_t\ |\ \exists s' \in R_t \wedge (s', t') \in E^\uparrow \} \text { and } T = {(T^\uparrow )}^* $$

where $E^\uparrow$ is the set of back edges, $*$ is the reflexive and transitive closure.

### Online Part

The online part leverages precomputed and dominance information to answer liveness queries efficiently. Boissinot et al.’s algorithm ([7, Algorithm 1]) is reproduced as Algorithm 1. Given a variable a and a program point *q*, the algorithm filters the content of $T_q$ to keep only the set $T_{(q, \texttt {a})}$ of points that are strictly dominated by the definition point of a (line 2). Then it tests whether one of these points can reach a use of a in the reduced graph (lines 3–4). If one test succeeds, then it returns $true$ (line 4), the variable a is live at *q*, otherwise it returns $false$ (line 5), the variable a is not live at *q*. In Fig. 2a, $T_{(5, \texttt {x}_1)} = \{ 4, 5 \}$, $uses (\texttt {x}_1)=\{4,9\}$, $4 \in R_4$, thus $\texttt {x}_1$ is live at 5. $T_{(5, \texttt {x}_2)} = \emptyset$, thus $\texttt {x}_2$ is not live at 5. $T_{10} = \{ 3, 10 \}$, $T_{(10, \texttt {x}_1)} = \{ 10 \}$, $R_{10} \cap uses (\texttt {x}_1) = \emptyset$, thus $\texttt {x}_1$ is not live at 10.
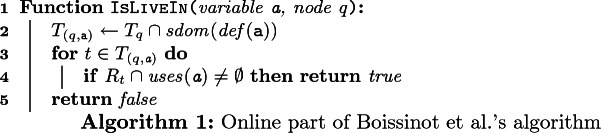


## Formalization

Our Coq implementation follows approximately the same structure as the algorithm described in Sect. 2. In particular, it is divided into two parts: the precomputation and the online parts.

### Precomputation

As highlighted in Sect. 2.2, the precomputation step depends only on the CFG structure. Thus, we can abstract the specific features of the SSA form and only work at the graph-theoretic level. We model the CFG as a map of type  2 associating to each node the list of its successors, and a node  representing the entry point of the CFG. Moreover, to implement the second optimization described in Sect. 3.2, we need to model the preorder numbering on the dominance tree. We assume that we are given a function  associating to each node the corresponding number.

As proposed in [7], the precomputation step itself is split into two parts. In [7], the first one computes *R*, while the second one computes *T* based on *R*. We slightly adapted both parts. In our implementation, the first part computes *R* and $T^\uparrow$, and the second part computes *T* in a different way than in [7].

**Fig. 4.**
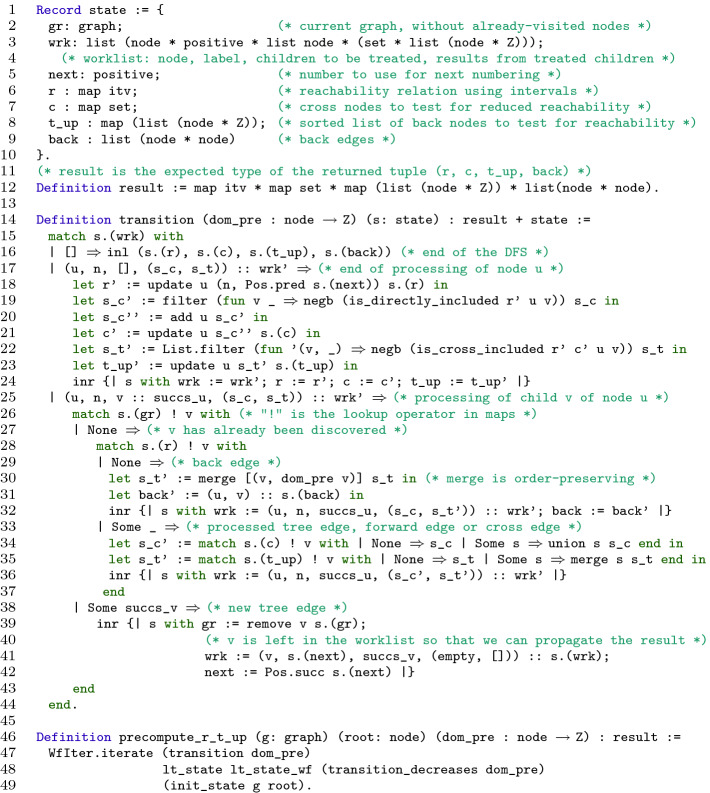
Function  implements the first part of the precomputation.

**Precomputation of** $\varvec{R}$ **and** $\varvec{T^\uparrow }$**.** Boissinot et al. [7] suggest encoding the set of reduced reachable nodes from node *t*, $R_t$, as a set (using bitsets or sorted arrays). But they assume, as is the case for most compilers, that the nodes in the CFG represent blocks of instructions, which means that the CFG is not really large. CompCertSSA’s peculiarity is that, like CompCert, each node in the CFG represents only one instruction, and thus the CFG is noticeably bigger. To avoid manipulating large sets, we decided to encode *R* differently, drawing our inspiration from Boissinot et al.’s idea to treat back edges specially. We choose to treat cross edges specially, and to break down reachability in the reduced graph into reachability in the spanning tree from sets of cross edge targets. This decomposition seems to forget forward edges, but as far as only reachability is concerned, they can be safely ignored, as they are just shortcuts of tree edges.

We introduce the relations $\widetilde{R}$ that denotes the reachability in the spanning tree, and *C* that associates to each program point both itself and a set of cross edge targets that are interesting for checking reduced reachability at this point. Like *T*, *C* is defined as the reflexive and transitive closure of $C^\uparrow$, where $C^\uparrow _t$ (cf. Definition 2) associates to node *t* the set of cross edge targets not tree reachable (i.e. reachable in the spanning tree) from *t* but whose source is tree reachable from *t*. In Fig. 2a, only $C^\uparrow _8 = \{ 7 \}$ is non-empty. We have thus $C_8 = \{ 7, 8 \}$.

#### Definition 2

**(**$$C^\uparrow $$ **and** *C***).**

$$ C^\uparrow _t = \{ t' \in V \setminus \widetilde{R}_t\ |\ \exists s' \in \widetilde{R}_t \wedge (s', t') \in \widetilde{E}^\uparrow \} \text { and } C = {(C^\uparrow )}^* $$

where $\widetilde{E}^\uparrow$ designates the set of cross edges.

Moreover, since the spanning tree is a tree, we can use the technique mentioned in Sect. 2.1, i.e. encode $\widetilde{R}$ as a labeling of each node in the spanning tree with a pair of integers representing an interval. We can then answer reachability queries in the spanning tree efficiently by testing inclusion of those intervals.

In the Coq development, function  , shown in Fig. 4, implements this first step of precomputation. For the sake of clarity, the Coq code was a little prettified. In particular, the notation  , allowing to update only some fields of a record, is not a valid Coq expression.  returns a quadruple  , where:

encodes $\widetilde{R}$ by associating to each node an interval of  ;
3 implements *C*;
encodes $T^\uparrow$ (  associates to each node a list of pairs  where  is a node and  is just  ; this list is sorted on the second component (see Sect. 3.2); that second component is not really needed, it is a slight optimization that allows to reduce the number of calls to  by storing its result next to  the first time it is called);
is the list of identified back edges.

Function  performs a DFS traversal of the CFG. In the style of module Postorder of CompCert, it calls iteratively a transition function (l. 47) that updates a state (initialized l. 49) with the guarantee that the iterations eventually terminate (l. 48). The state aggregates seven fields (l. 1). Four fields (  ,  ,  and  ) correspond to the final results. The three other fields are used to implement the DFS:  remembers whether a node has already been seen during the traversal;  is the current value of the counter used to number the encountered nodes; and  is a worklist of nodes to be treated. Each element of  is a quadruple  , where  is a node labeled with number  ,  is the list of successors of  yet to be treated, and  and  (detailed below) are pieces of information, retrieved from the successors of  that have already been treated, and used to compute the value attached to  in  and  respectively.

Function  begins with checking whether the worklist is empty. If so (l. 16), it is the last iteration and the appropriate fields of the state are returned. If not, it analyzes the status of the first node  of the worklist. If it has still children to be treated (l. 25), it checks the status of the first child  . If  is new to the DFS (l. 38), it is given number  , and is explored recursively by extending the worklist (l. 41). If  has already been seen before during the DFS (l. 27), we retrieve from it the pieces of information that need to be propagated to  , and we update  and  accordingly depending on the type of edge connecting  and  (ll. 28–37). Note that, in the first case (l. 38),  is intentionally left as a child of  in the worklist (l. 41), so that it can be seen again in the second case (l. 33), and results can be propagated from  to  . If all the children of  (l. 17) have been treated, we use the data available in the state and the worklist to update maps  ,  and  at key  .

To update  , we attach to  (l. 18) an interval based on the number  associated to  when it was discovered (l. 41) and the current value of the counter  . The update of  relies on the following equation: $C_u = \{ u \} \cup \left[ \bigcup _{(u, v) \in \widetilde{E}} C_v \right] \setminus \widetilde{R}_u$. $C_u$ is computed from the sets $C_v$ of its children in the reduced graph (i.e. children *v* where (*u*, *v*) is not a back edge). The union of these sets (l. 34) is filtered (l. 19), so that only nodes that are not already tree reachable from *u* are kept. Finally, node *u* is added to the set (l. 20). The update of  relies on a similar equation: $T^\uparrow _u = \left[ \bigcup _{(u, v) \in E^\uparrow } \{ v \} \cup \bigcup _{(u, v) \in \widetilde{E}} T^\uparrow _v \right] \setminus R_u$. $T^\uparrow _u$ is computed from its children in the graph. If (*u*, *v*) is a back edge, then the contribution of *v* is $\{v \}$ (l. 30). If (*u*, *v*) is not a back edge, then the contribution of *v* is $T^\uparrow _v$ (l. 35). These sets are merged in an order-preserving way, and then filtered so that only nodes that are not already reduced reachable from *u* are kept (l. 22).

The edges in  are classically identified during the DFS (l. 31) as the edges from the current node to nodes already discovered but not fully processed.

In terms of structure, our code is really close to the code of module Postorder. There are two key differences, though. The first one is that we need to remember some information between the time a node is discovered and the time it is fully processed (the preorder number  ). The second one is that we need to propagate some information during the traversal (the sets  and  ). This implied the two following changes. Firstly, the tuples in our worklist are more complex, since they contain the additional data. In Postorder, the worklist has the simpler type  . Secondly, as mentioned above, a node that is discovered is left in the worklist as a child of its parent, so that some information can be propagated to its parent the second time it is seen.

**Precomputation of** $\varvec{T}$**.** The second part of the precomputation consists in computing *T* from $T^\uparrow$, i.e. computing the reflexive and transitive closure of $T^\uparrow$. For this, we follow another suggestion from Boissinot et al. consisting in using the following equation ([7, Equation (1)]): $T_v = \{ v \} \cup \left[ \bigcup _{w \in T^\uparrow _v} T_w \right]$, that we also call Equation (1). They note that, given a node *t*, all nodes $t'$ in $T^\uparrow _t$ have a DFS preorder number^4^ smaller than that of *t*. This means that if we treat the back edge targets by growing DFS preorder number, we can use this equation to compute *T* for all the back edge targets.

In our Coq development, this step is performed by  . It takes as arguments  , the preorder numbering on the dominance tree,  , the DFS preorder number, and  and  , returned by the previous step. It extracts the back edge targets from  , sorts them according to  , and uses Equation (1) to compute *T* for the back edge targets. It returns a map  which is  updated with the new values for the back edge targets. We are careful to preserve in  the sorting of the values of  according to  .

Boissinot et al. also suggest computing *T* for the rest of the nodes by traversing the reduced graph in a second phase. Instead, we choose to use the same equation. This is the role of function  . It takes as an argument  and the map  returned by  , and applies Equation (1) to every node in any arbitrary order. This means that we also apply it to back edge targets, though they already have the right value, but this is correct and probably not costly. As before, we take care to ensure that the values of the returned map,  , are sorted according to  . However, we drop the preorder number component from the elements of  . They are no longer necessary, and, as mentioned in Sect. 3, were only there as an optimization.

Finally, function  assembles both previous functions to compute *T* from $T^\uparrow$.
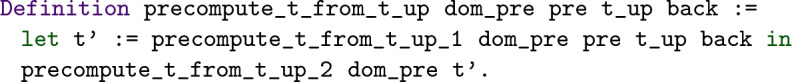


**Assembling.** To obtain the full precomputation step, we just have to assemble the pieces introduced in the previous sections. This is the role of  .
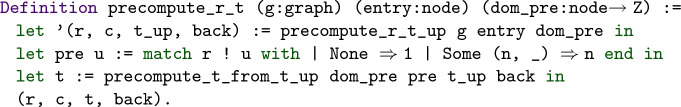


It takes as arguments a graph  , an entry node  and a preorder numbering on the dominance tree,  . It returns *R* (encoded as  and  ), *T* (encoded as  ) and the list of back edges,  . Note that  , the DFS preorder number, is simply defined as a lookup in  .

### Online Part

The implementation of the online part in Coq is faithful to Algorithm 1, but also takes advantage of optimizations discussed in [7]. More precisely, it is an adaptation of [7, Algorithm 3] that uses sorted lists instead of bitsets, and functional instead of imperative programming.

Indeed, Boissinot et al. suggest two optimizations to speed up Algorithm 1. The first one, that we call **(opt1)**, consists in testing at the beginning whether *q* is strictly dominated by the definition point of a. If that is not the case, as explained in Sect. 2.3, $false$ can be returned immediately. The second one, denoted **(opt2)**, uses dominance information more. The idea is that if we test a node *t* in $T_{(q, \texttt {a})}$ and that fails, then the test for any $t'$ dominated by *t* will fail too, and thus we can skip all such nodes. For instance, in Fig. 2a, $T_{(5, \texttt {x}_0)} = \{ 3, 4, 5 \}$, $R_3 \cap uses (\texttt {x}_0) = \emptyset$, and 3 dominates 4 and 5, thus we can return $false$ without testing 4 and 5. Boissinot et al. suggest taking advantage of a preorder numbering on the dominance tree. This numbering can be used in two ways. It can be used to sort $T_q$, since the node with the lowest number is likely to dominate the other nodes to be tested (this is always the case if the CFG is reducible). It can also be used as described in Sect. 2.1, to build a dominance test in constant time.

**Fig. 5.**
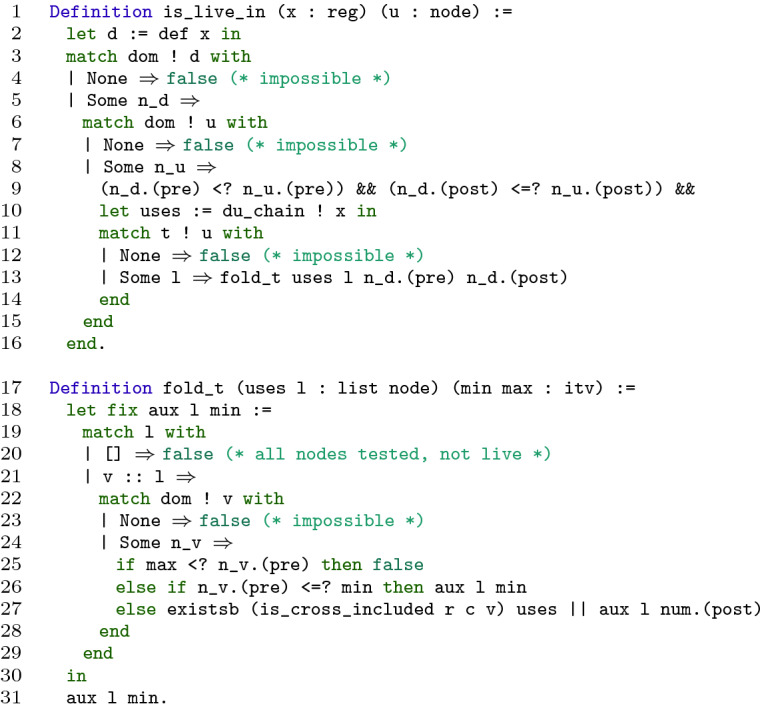
Function  implements the online part of the algorithm.

Our implementation is parameterized by the following objects.  associates to each node an interval based on its preorder number in the dominance tree (this numbering is actually used to implement  in the precomputation step, cf. Sect. 3.1);  associates to each variable of type  its definition point;  connects each variable to the points where it is used;  ,  and  are the results of the precomputation part. Based on these objects, we implement function  , given in Fig. 5.  returns whether variable  is live at point  . It is a bit difficult to read due to Coq syntax and notations, but it is rather straightforward.

First, we get the definition point,  , of variable  (l. 1). Then we get the preorder intervals in the dominance tree of  and  (ll. 3–8). We check that the interval of  is strictly included in that of  (l. 9), meaning that  is strictly dominated by  , otherwise we directly return  (this is **(opt1)**). Then we get the list  of program points where  is used (l. 10), and we read in  the list  of points to test to answer the liveness query (l. 11). Recall that  is sorted according to the preorder numbering on the dominance tree. Then we call  that tests the nodes in  one after the other.

 performs case analysis on  . If it is empty (l. 20), this means that we have tested all the nodes and none of them have revealed a path to a use of  , thus we return  . Else, we consider the first element  of  (l. 21) and its preorder interval  in the dominance tree (l. 22). If  , the left bound of the interval  , is greater than  (l. 25), this means that  is not dominated by  , and neither are the other nodes in  , thus we can answer  . Otherwise, if  is not larger than  (l. 26), this means that  is not strictly dominated by  or is dominated by a node that has been tested unsuccessfully in a previous iteration, thus we can skip  . Otherwise (l. 27), we test if a node in  is reduced reachable from  thanks to function  . If yes, we return  . Otherwise, we test the other nodes of  and update the minimal bound to  , the right bound of the interval  , so that nodes dominated by  are skipped in the next iterations.

## Proof of Correctness

The functions described in Sect. 3 all come with proofs of their correctness. However, among the pieces of CompCertSSA on which we build our work, one, namely the formalization of the dominance test [4], turned out to be too weak for our purposes. Indeed, it is proved correct, but not complete, while its completeness is necessary to prove the correctness of our approach. There is an ongoing effort based on [10] to build a correct and complete dominance test, but for now, completeness is admitted.

Most of the proof effort lies in the precomputation part (  , 1700 lines of specification and 4000 lines of proof), and especially in the proof of  that required dozens of invariants. While this number could undoubtedly be decreased, it shows that the justification of the operations performed during the DFS is non-trivial.

For lack of space, we do not detail the proofs of  and  . We just want to emphasize one point in the proof of the latter.  is written using a  operation on the list of back-edge targets, and the validity of this computation is really subtle. Indeed, it relies on Equation (1) and the fact that nodes are considered in the right order, i.e. in increasing DFS preorder number. To ease the definition of complex invariants, we reuse the architecture of  (cf. Fig. 4), but this time only on the proof side. This form allows to express more easily properties involving the nodes that have already been processed or those that are to be processed. We then show the equivalence of this form with the  -based version, and we conclude about the correctness of  .

To state the correctness theorems of  , we assume we are given a graph  , a node  in  , and a labeling function  . We make two reasonable assumptions about  and  . 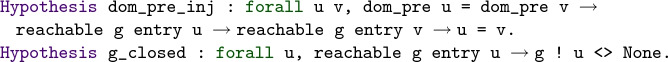  ensures that the preorder numbering on the dominance tree modeled by  is injective.  ensures a kind of well-formedness of  , namely that all nodes reachable from  must be in  .

We can note that both hypotheses take as preconditions that the considered nodes are reachable from the entry node of the CFG. Actually, most of the results have this kind of hypothesis, since the DFS from node  can only discover nodes reachable from  . In this section, such hypotheses will appear in the formal statements, but we will ignore them in the discussion.

Under these hypotheses, we can state the two main correctness theorems of  . They state that it computes correctly relations *R* and *T*. 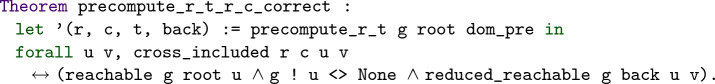  states that a node  is reduced reachable from a node  if and only if  and  are related by predicate  , meaning that  is tree reachable from one node in $C_u$. 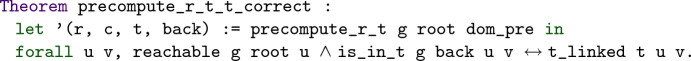  states that a node  is in $T_u$ (modeled by  ) if and only if  is in the list associated to  in  (specified by  ).

The proof of correctness of the online part is much smaller (230 lines of specification, 1000 lines of proof). One big fragment of it is the proof of the link between *T* and the existence of strictly dominated paths, that justifies the use of *T* in the liveness analysis.  is a lemma from this fragment. It states that if  is a strictly  -dominated path between  and  , then there exists a node  in $T_u$, strictly  -dominated and from which *v* is reduced reachable. 
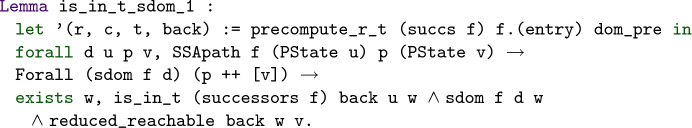
 The proof of this lemma is interesting, because the proof given by Boissinot et al. in [7] was not easily translatable in Coq. Indeed, their proof consists in considering a path with a minimal number of back edges among the strictly *d*-dominated paths from *u* to *v*. Such a property is not easy to express in Coq. We proved this result in another manner, by induction on the path.

Finally, theorem  states the correctness of the liveness analysis, namely that if the analysis succeeds, a liveness query is answered $true$ if and only if the considered variable is live at the considered program point.  is a predicate guaranteeing that function  is well-formed. It allows to prove the hypotheses of the lemma described above (e.g.  ). 
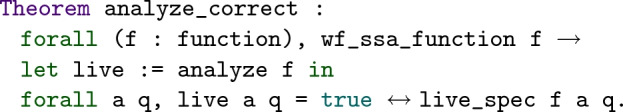


## Experiments

To evaluate the efficiency of the liveness checking approach, we compare it experimentally w.r.t. a standard liveness analysis.

More precisely, our reference implementation, called **(impl1)**, is a standard analysis based on data-flow equations. As already mentioned, CompCertSSA contains several liveness analyses, but actually none of them are defined on SSA, so we adapted one of them to SSA. Like the existing ones, this analysis uses the data-flow solver provided by CompCert in module  , but takes into account the particularities of SSA, especially the $\phi$-nodes.

The two other implementations, called **(impl2)** and **(impl3)**, are variations of the implementation presented in Sect. 3. They both implement **(opt1)** mentioned in Sect. 3.2. However, **(impl2)** implements **(opt2)** only partially, it only sorts the nodes in $T_q$ by their preorder number in the dominance tree, while **(impl3)** implements it fully, since it can also skip a subtree of the dominance tree when a test fails.

We ran the three implementations on a set of programs taken from CompCert’s benchmarks. These programs cover a wide range of size. Most of these programs are one or a few hundred lines long, some of them (e.g. bzip2 and raytracer) are a few thousand lines long, and one of them (spass) contains more than 50,000 lines. Experiments were conducted on a Dell Latitude 7490 with an Intel Core i7-8650U processor at 1.90 GHz and 16 GB of memory.

To perform the comparison, we need a set of liveness queries. To generate these, the best option would be to use a real compiler pass relying on liveness. However, CompCertSSA does not include such a pass at the level of SSA. We came up with the following, admittedly contrived, solution. We generate one query per variable and per natural loop header (a node dominating one of its predecessors). We do not know whether this kind of query is representative of actual queries. However, we can verify that the number of queries is reasonable. In particular, we have two programs in common with Boissinot et al.’s benchmarks: bzip2 and mcf. On both programs, we ask more queries (bzip2: 275071 vs. 10100, mcf: 3748 vs 2369). As doing too many queries penalizes us, the results we give underestimate the benefits of our implementation. Yet, this way of generating queries is fundamentally biased, since depending on the number of loops in a function, the number of queries varies widely. In particular, the functions with no loops are not tested. One program (fib) even has no loop, thus no query. We thus removed it from the experiments.

**Fig. 6.**
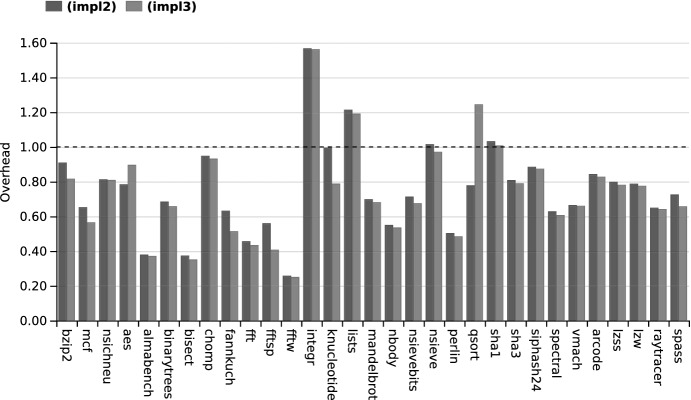
Total overhead of **(impl2)** and **(impl3)** w.r.t. **(impl1)**

We first compared separately the precomputation and online parts of **(impl2)** and **(impl3)** w.r.t. **(impl1)**. The results, not included in the paper for lack of space, but available in [1], confirm the expected trends: **(impl1)** is significantly slower than **(impl2)** and **(impl3)** in the precomputation part, and significantly faster in the online part. Then, we compared the total time taken by both parts performed successively in **(impl2)** and **(impl3)** w.r.t. the time they take in **(impl1)** (see Fig. 6). We can observe that **(impl2)** and **(impl3)** are in nearly all the cases faster than **(impl1)**. With the set of queries considered, liveness checking is thus a better trade-off than standard liveness analysis in terms of efficiency. If we compare our results to those obtained by Boissinot et al. [7], we observe a better average speedup (1.48, with **(impl3)**) of liveness checking w.r.t. standard liveness than them (1.16). But there are many differences in terms of implementation and testing process between Boissinot et al.’s work and ours, thus the comparison of these numbers is of limited value. On the comparison of **(impl2)** and **(impl3)**, we can notice that **(impl3)** is in almost all cases faster than **(impl2)**, although moderately, showing that the added complexity of **(impl3)** is worthwhile. There are two exceptions, aes and qsort, but with no clear explanation.

## Conclusion and Perspectives

We have described the formalization and implementation in the CompCertSSA verified compiler of the liveness analysis described in [7]. This analysis belongs to the “liveness checking” category, i.e. it is designed to answer liveness queries of the form “is variable a live at point *q*?”. Its proof of correctness involves the combination of non-trivial arguments about liveness, SSA form, dominance and depth first search. Limited experiments show that, as expected, this algorithm outperforms the classic data-flow based approach if the number of queries is low.

Boissinot et al.’s work is not the only alternative to the data-flow based technique. Appel [2] describes how to propagate liveness information backwards from uses to definitions in programs in SSA form. Boissinot et al. [5] extended the ideas of [7] in 2011, still for SSA-form programs, by taking advantage of an auxiliary structure called a loop-nesting forest. They also propose two variants of Appel’s approach, and experimentally compare the three algorithms. Das et al. suggest DJ-graphs rather than loop-nesting forests as auxiliary structures. Among all these works, only [7] and Das et al. [8] embrace the “liveness checking” approach.

One limitation of this work is that it has not been used in a real pass of CompCertSSA yet. This is the reason why we came up with an artificial criterion to evaluate our approach. One pass where it could be used is SSA destruction. Indeed, Boissinot et al. detail in yet another work [6] an SSA destruction pass that uses liveness checking. We could take advantage of [9] that already formalized most of [6] in CompCertSSA, but used a traditional data-flow-based liveness analysis. However, [6] describes an approach with a linear number of queries, while, for the sake of simplicity, [9] makes a quadratic number of them. As the “liveness checking” approach is interesting only if the number of queries is low, we would need to implement the clever approach of [6] first.

A natural extension of this work is the mechanization of Boissinot et al.’s algorithm based on loop-nesting forests [5]. The formalization of a such a complex structure would certainly add a level of difficulty to the correctness proof, but this structure is generic enough to serve as a basis for other program analyses and transformations (e.g. [14]), thus formalizing it could turn out to be profitable.

## Footnotes

1 Given a relation *R*, $R_x$ denotes the set of elements related to *x* in *R*.

2 
 is an alias for  , a binary encoding of strictly positive integers;  is implemented using  , an associative map whose keys are  and which is used pervasively in CompCertSSA.

3 
 is a map where only keys are meaningful.

4 This numbering must not be confused with  , the preorder numbering on the dominance tree.
